# Use of Retrieval-Augmented Large Language Model for COVID-19 Fact-Checking: Development and Usability Study

**DOI:** 10.2196/66098

**Published:** 2025-04-30

**Authors:** Hai Li, Jingyi Huang, Mengmeng Ji, Yuyi Yang, Ruopeng An

**Affiliations:** 1 School of Economics and Management Shanghai University of Sport Shanghai China; 2 Department of Surgery Division of Public Health Sciences Washington University School of Medicine in St. Louis St. Louis, MO United States; 3 Division of Computational and Data Sciences Washington University in St. Louis St. Louis, MO United States; 4 Constance and Martin Silver Center on Data Science and Social Equity Silver School of Social Work New York University New York, NY United States

**Keywords:** large language model, misinformation, disinformation, fact-checking, COVID-19, artificial intelligence, ChatGPT, natural language processing, machine learning, SARS-CoV-2, coronavirus, respiratory, infectious, pulmonary, pandemic, infodemic, retrieval-augmented generation, accuracy

## Abstract

**Background:**

The COVID-19 pandemic has been accompanied by an “infodemic,” where the rapid spread of misinformation has exacerbated public health challenges. Traditional fact-checking methods, though effective, are time-consuming and resource-intensive, limiting their ability to combat misinformation at scale. Large language models (LLMs) such as GPT-4 offer a more scalable solution, but their susceptibility to generating hallucinations—plausible yet incorrect information—compromises their reliability.

**Objective:**

This study aims to enhance the accuracy and reliability of COVID-19 fact-checking by integrating a retrieval-augmented generation (RAG) system with LLMs, specifically addressing the limitations of hallucination and context inaccuracy inherent in stand-alone LLMs.

**Methods:**

We constructed a context dataset comprising approximately 130,000 peer-reviewed papers related to COVID-19 from PubMed and Scopus. This dataset was integrated with GPT-4 to develop multiple RAG-enhanced models: the naïve RAG, Lord of the Retrievers (LOTR)–RAG, corrective RAG (CRAG), and self-RAG (SRAG). The RAG systems were designed to retrieve relevant external information, which was then embedded and indexed in a vector store for similarity searches. One real-world dataset and one synthesized dataset, each containing 500 claims, were used to evaluate the performance of these models. Each model’s accuracy, *F*_1_-score, precision, and sensitivity were compared to assess their effectiveness in reducing hallucination and improving fact-checking accuracy.

**Results:**

The baseline GPT-4 model achieved an accuracy of 0.856 on the real-world dataset. The naïve RAG model improved this to 0.946, while the LOTR-RAG model further increased accuracy to 0.951. The CRAG and SRAG models outperformed all others, achieving accuracies of 0.972 and 0.973, respectively. The baseline GPT-4 model reached an accuracy of 0.960 on the synthesized dataset. The naïve RAG model increased this to 0.972, and the LOTR-RAG, CRAG, and SRAG models achieved an accuracy of 0.978. These findings demonstrate that the RAG-enhanced models consistently maintained high accuracy levels, closely mirroring ground-truth labels and significantly reducing hallucinations. The CRAG and SRAG models also provided more detailed and contextually accurate explanations, further establishing the superiority of agentic RAG frameworks in delivering reliable and precise fact-checking outputs across diverse datasets.

**Conclusions:**

The integration of RAG systems with LLMs substantially improves the accuracy and contextual relevance of automated fact-checking. By reducing hallucinations and enhancing transparency by citing retrieved sources, this method holds significant promise for rapid, reliable information verification to combat misinformation during public health crises.

## Introduction

The COVID-19 pandemic has been accompanied by an “infodemic,” characterized by the rapid spread of misinformation and disinformation, significantly undermining public health efforts [1]. Misinformation refers to the unintentional spread of incorrect or misleading information [2], while disinformation involves deliberately disseminating false information to deceive [3]. Both phenomena contribute to disseminating inaccurate health information during crises such as the COVID-19 pandemic, leading to high-risk behaviors and overwhelming public health systems [4].

Fact-checking is crucial to combat health misinformation and disinformation. During the early stages of COVID-19, from January to March 2020, fact-checking related to COVID-19 increased by over 900% [5]. This fact-checking primarily addressed topics such as illness, transmission and mortality rates, control measures, treatment options, and the causes of the disease [6]. For example, rapid and widespread claims regarding substances such as lemon or bleach as cures for COVID-19 required prompt fact-checking to prevent adverse consequences.

Conventional COVID-19 fact-checking methods have several notable drawbacks [7]. Obtaining fact-checking directly from human experts or scientists can be time-consuming and challenging. The emergence of tools based on large language models (LLMs), such as OpenAI’s ChatGPT, has the potential to offer more efficient and convenient COVID-19 fact-checking than human checking. However, they also tend to generate plausible but incorrect or nonsensical information not grounded in the input data or real-world knowledge, commonly referred to as hallucinations [8,9]. Hallucinations can occur due to several reasons. First, LLMs may generate incorrect information because they rely heavily on the patterns and associations found in their training data, which can be subject to biases, inaccuracies, and gaps in the original data sources [10]. Second, LLMs lack real-world understanding and contextual awareness, meaning they do not possess the ability to verify facts against actual events or comprehend nuanced contexts [11]. In addition, the inherent probabilistic nature of LLMs can result in generating outputs that are statistically plausible but factually incorrect [12]. Hallucinations significantly undermine the reliability of LLMs in identifying and correcting disinformation and misinformation, posing a severe challenge to their effectiveness in safeguarding public health information during the COVID-19 pandemic.

Retrieval-augmented generation (RAG) is a state-of-the-art technique that enhances LLMs by integrating external data retrieval, improving factual accuracy, and reducing costs [13]. By retrieving relevant information from external sources and incorporating it as contextual input, RAG effectively mitigates the issue of hallucinations in LLMs [14]. This method also addresses the need for updated external knowledge without requiring costly training or fine-tuning, making it a practical solution for maintaining the reliability and relevance of LLMs in dynamic information environments [15].

This study aims to evaluate the effectiveness of the RAG system in enhancing the performance of LLMs for COVID-19 fact-checking. By integrating a contextual dataset of about 130,000 peer-reviewed papers on COVID-19 with GPT-4 as the base model, we assessed the performance of various RAG-equipped models. Our research contributions follow. First, we developed a novel RAG-equipped LLMs method that significantly reduces both financial and time costs compared with conventional COVID-19 fact-checking approaches. Second, RAG-equipped LLMs enhance the reliability of COVID-19 fact-checking by providing specific explanations and references from peer-reviewed journals, effectively reducing hallucinations and external factual errors in LLMs. Finally, based on these promising results, this study could pave the way for developing a targeted web application for COVID-19 fact-checking designed to improve individuals’ understanding of accurate health information and support public health initiatives in combating misinformation.

## Methods

### Data

First, we constructed a context dataset for the RAG system by searching for “COVID-19” and “SARS-CoV-2” within the PubMed and Scopus databases. This dataset comprises 126,984 papers published in peer-reviewed journals between January 1, 2020, and January 1, 2024. About 31% (39,365/126,984) of the included papers were published in 2021 or 2022, and 22% (27,936/126,984) were published in 2023, which ensured that the dataset includes up-to-date and matured insights into COVID-19. Our RAG system leveraged this dataset as external knowledge to provide reliable and accurate COVID-19 fact-checking based on academic references.

To evaluate the RAG system, we compiled two datasets of COVID-19–related claims (Multimedia Appendix 1). The first dataset comprises 500 claims from social media posts and news outlets obtained via Kaggle (founded in 2010 by Anthony Goldbloom and Jeremy Howard and acquired by Google in 2017). The second dataset consists of 500 synthesized claims generated by GPT-4 using the context dataset of peer-reviewed academic papers. Both datasets were balanced, with 50% of the claims being true and 50% false. These datasets were used to access model performance. Table 1 provides examples of both true and false claims.

**Table 1:** Examples of claims of COVID-19.

Type and example claims of COVID-19			Label
**Real-world dataset**			
	Drinking methanol, ethanol, or bleach will not prevent or cure COVID-19 and can be very dangerous.	True	
	Most people who contract COVID-19 will recover.	True	
	Avocado and mint tea cure coronavirus.	False	
	Chewing raw onions can prevent COVID-19.	False	
**Synthesized dataset**			
	Antifibrotic therapies are being considered to manage progressive pulmonary fibrosis observed in some severe cases of COVID-19.	True	
	Confusion or a sense of disorientation has been documented in patients with COVID-19, especially in older people.	True	
	Smoking and long-term exposure to second-hand smoke are not associated with increased susceptibility to acute COVID-19 infection.	False	
	IL-6^a^ receptor antagonists are ineffective in treating the cytokine release syndrome associated with COVID-19.	False	

^a^IL-6: Interleukin-6.

### Data Preprocessing

**Figure 1:**
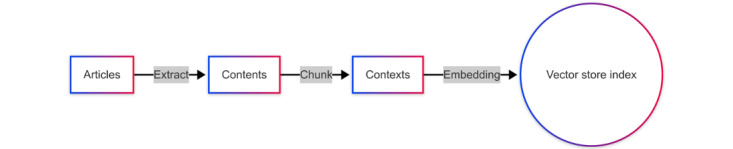
Workflow of data preprocessing.

Figure 1 illustrates the data preprocessing workflow for the RAG system. We extracted each academic paper’s abstract, title, authors, published journal, and publication date. Due to the well-structured nature of the academic paper’s abstract, we can ensure accuracy while avoiding copyright issues and minimizing computational costs using the extracted information rather than the full-text papers. The abstracts were segmented into smaller chunks with appropriate length and overlapping, which served as the primary input for further processing. The remaining details were treated as contextual metadata, which complements the abstract chunks during the retrieval process. Each segmented chunk was transformed into a numerical format known as a vector. These vectors are mathematical representations that enable computational operations. Vectors were stored in the vector store index within the Qdrant database, which was designed for similarity searches. The system used maximal marginal relevance technology to optimize the retrieval process by balancing relevance and diversity in the retrieved results.

### Model Architecture

#### Base Model

We used OpenAI’s GPT-4 as the foundational model for the RAG system. In the corrective RAG (CRAG) and self-RAG (SRAG) workflow, GPT-4 additionally functions as a grader or rewriter model.

#### Embedding Models

We used two distinct embedding models in the RAG systems. The “text-embedding-ada-002” model provided by OpenAI was used across all RAG systems, and the “NeuML/pubmedbert-base-embeddings” model from Hugging Face was specifically applied in the LOTR-RAG system.

#### Naïve RAG System

The naïve RAG system combines the parametric memory of a pretrained LLM with the nonparametric memory of a vector index containing specific external knowledge [13].

**Figure 2:**
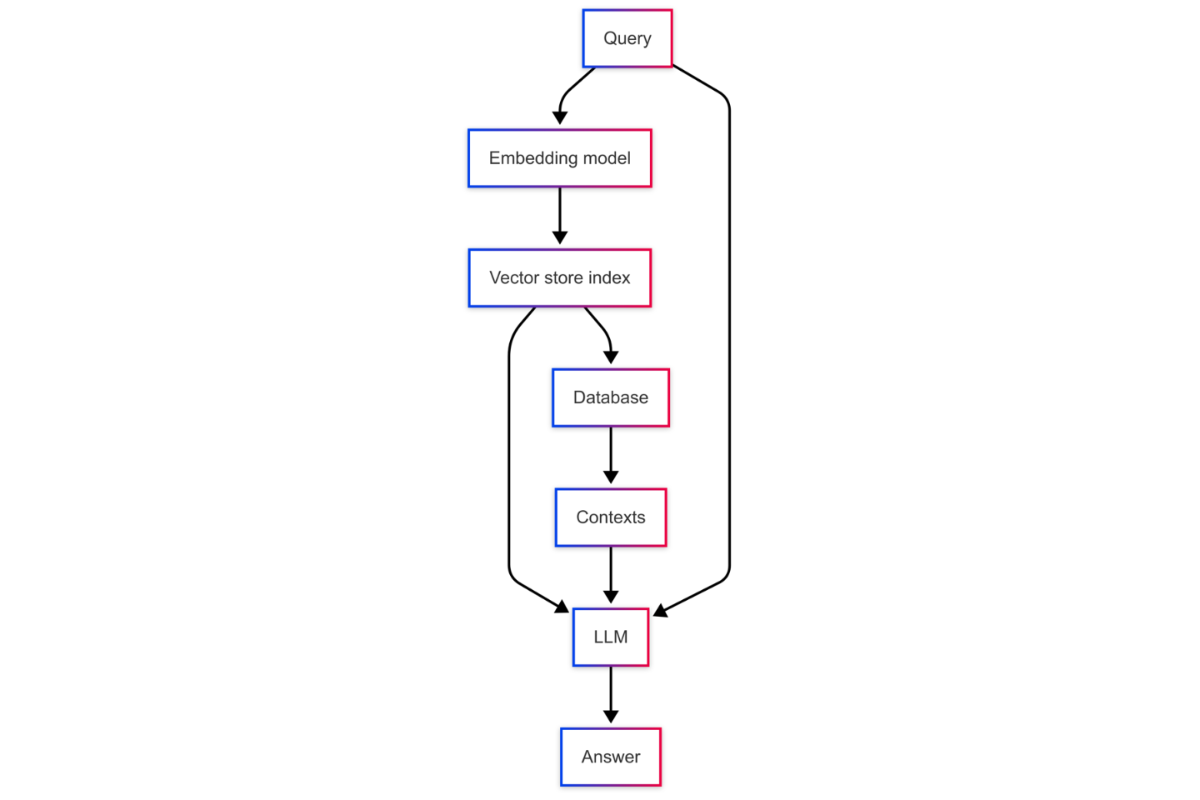
Workflow of naïve retrieval-augmented generation. LLM: large language model.

Figure 2 shows that the naïve RAG workflow begins with embedding the input query into a vector, followed by a similarity search within the vector store to retrieve the most relevant contexts. The LLM then conducts fact-checking on each COVID-19 claim based on these retrieved contexts, ensuring information retrieval accuracy and simultaneously providing reliable explanations and references.

Despite its effectiveness, the naïve RAG has limitations, such as the “lost in the middle” phenomenon, where the LLM’s performance deteriorates when handling information located in the middle of lengthy contexts [16]. To address this issue, we used the Lord of the Retrievers (LOTR)–RAG system [17].

#### LOTR-RAG System

**Figure 3:**
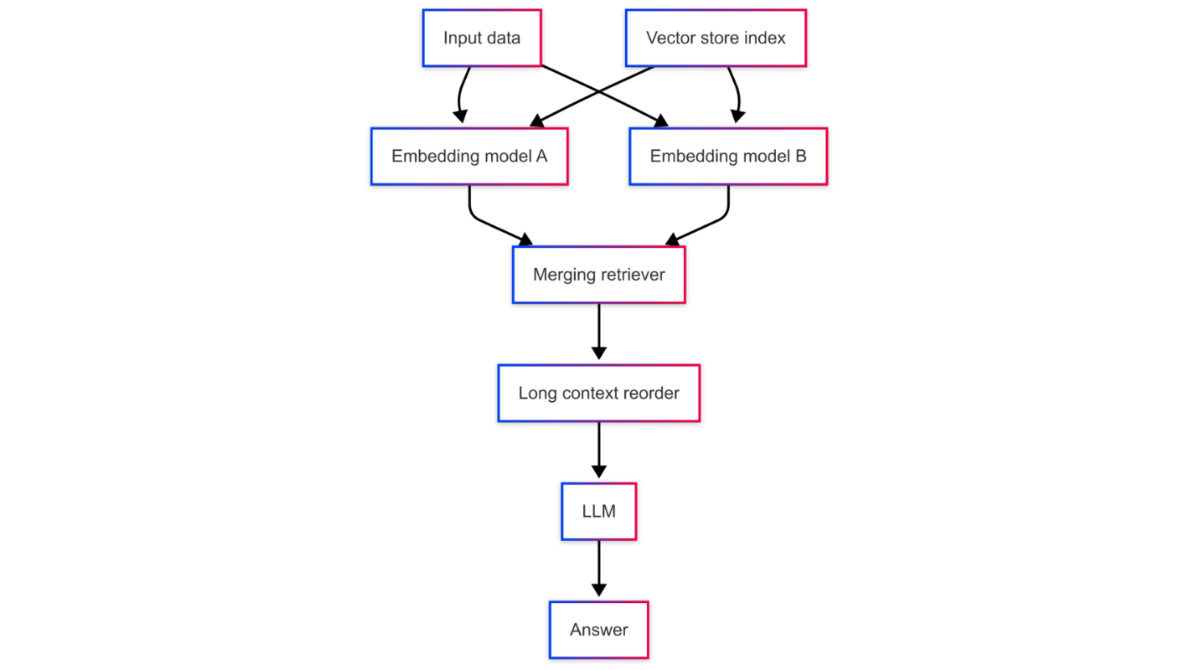
Workflow of Lord of the Retrievers–retrieval-augmented generation. LLM: large language model.

As Figure 3 illustrates, the LOTR-RAG system enhances the relevance between context and query by using a merging retriever that integrates two distinct embedding models. This approach allows for creating two vector store indices, each associated with our context dataset, leading to more coherent and accurate responses than the naïve RAG system [18].

However, for more complex tasks such as fact-checking, the LOTR-RAG system may still be limited by integrating a single LLM and RAG system [19]. To overcome these limitations, we introduced agentic frameworks—CRAG and SRAG—to further enhance the reliability of fact-checking.

#### CRAG System

**Figure 4:**
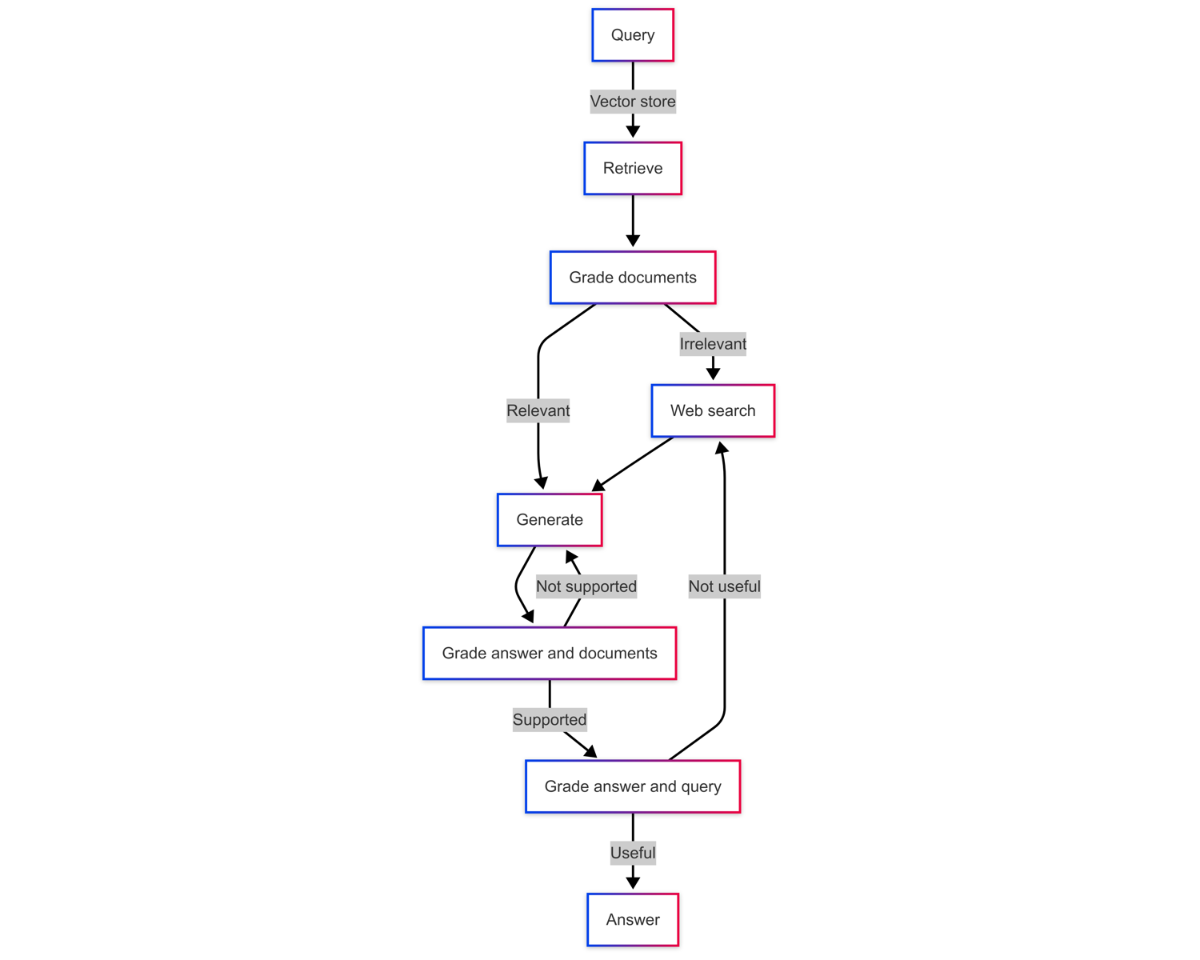
Workflow of corrective retrieval-augmented generation.

Figure 4 illustrates the CRAG [20] workflow. GPT-4 functions as a grader in this framework, evaluating the input, retrieval, and generation processes. The grader decides whether to retrieve information from the vector store or use the Tavily Search application programming interface, an LLM-optimized search engine developed by Tavily, which efficiently aggregates and ranks data from multiple sources for fast and consistent results [21]. During the retrieval process, the grader assesses the relevance of the documents, and irrelevant documents are replaced with those obtained via web search. After answer generation, the grader verifies whether the answer is helpful for the fact-checking task and supported by the retrieval documents. If not, additional information is retrieved, or the answer is regenerated until it meets the criteria.

#### SRAG System

**Figure 5:**
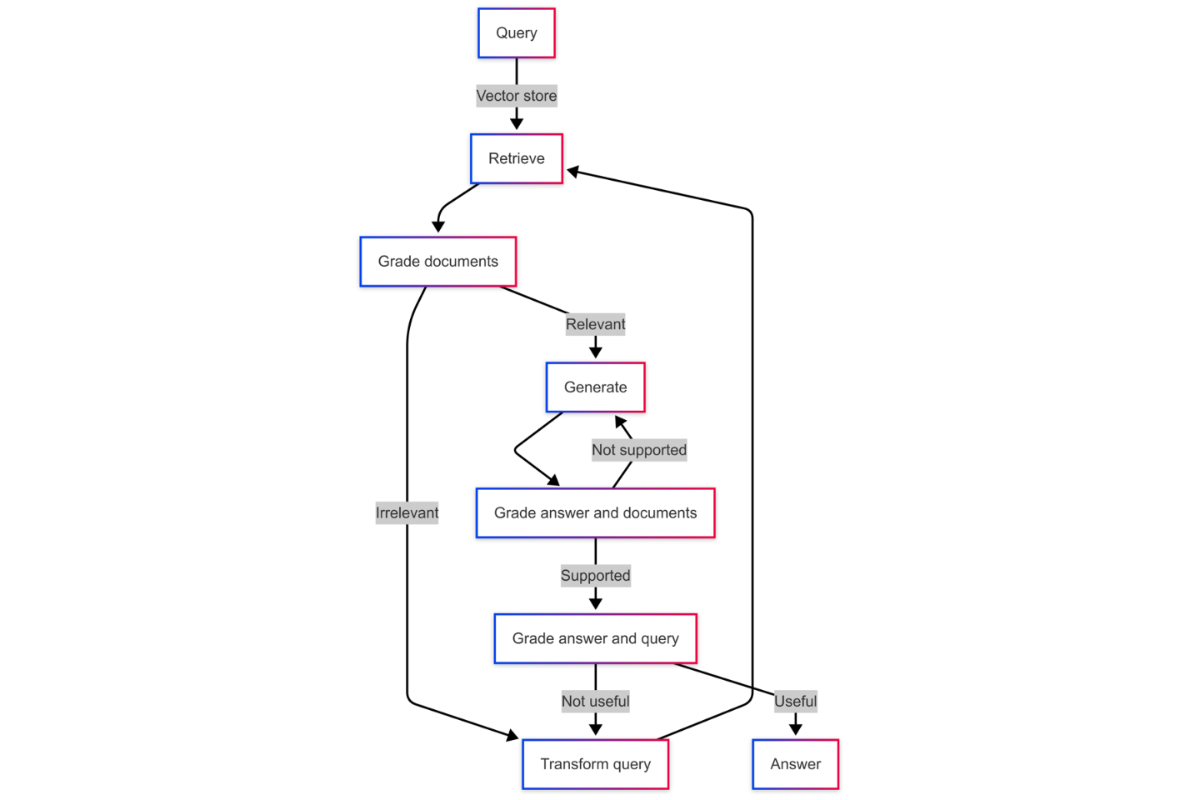
Workflow of self–retrieval-augmented generation.

Figure 5 illustrates the SRAG [22] framework, which uses GPT-4 as both grader and rewriter. After retrieving documents, the grader assesses their relevance to the original query. If the documents are deemed irrelevant, the rewriter reformulates the query while preserving its original semantic meaning to enhance retrieval effectiveness. This iterative process continues until the retrieved documents demonstrate high relevance to the query, at which point an answer is generated. Following answer generation, the grader evaluates whether the response adequately addresses the input query and is supported by the retrieved documents. If the answer does not meet these criteria, the framework either rewrites the query or regenerates the answer, repeating the process until a satisfactory and reliable response is achieved.

By integrating agentic frameworks such as CRAG and SRAG, the reliability of COVID-19 fact-checking is significantly enhanced, as the LLM’s outputs are rigorously evaluated before being presented to the user.

### Prompt Engineering

Table 2 outlines the prompts used in this study. For answer generation, the LLM is instructed to perform COVID-19 fact-checking based on retrieved documents from the context dataset, functioning as an expert. In the agentic frameworks of CRAG and SRAG, the LLM is further directed to evaluate the quality of retrieved documents and generated answers, ensuring the robustness of the entire workflow.

**Table 2:** Prompts using in experiments.

Objective	Prompt
Generate answers	You are an expert for the COVID-19 fact-checking tasks. Based on pieces of retrieved context to detect if the claim is true or false. You will have to give me the title and author of the context you referred to in one sentence. If you don’t know the answer, just say that you don’t know. Keep the answer concise. Claim: {question} Context: {context} Answer:
Grade documents	You are a grader assessing relevance of a retrieved document to a user question. If the document contains keywords related to the user question, grade it as relevant. It does not need to be a stringent test. The goal is to filter out erroneous retrievals. Give a binary score “yes” or “no” score to indicate whether the document is relevant to the question. Provide the binary score as a JSON with a single key “score” and no preamble or explanation. Here is the retrieved document: {document} Here is the user question: {question}
Grade answers	You are a grader assessing whether an answer is useful to resolve a question. Give a binary score “yes” or “no” to indicate whether the answer is useful to resolve a question. Provide the binary score as a JSON with a single key “score” and no preamble or explanation. Here is the answer: {generation} Here is the question: {question}
Rewrite claims	You are a claim rewriter that converts an input claim to a better version that is optimized for vector store retrieval and fact-checking. Look at the input and try to reason about the underlying semantic intent meaning.

### Ethical Considerations

This study used preexisting public data with no identifiable information, due to them being anonymized, and therefore does not require institutional review board review per Federal Regulations for the Protection of Human Research Subjects (45CFR 46.104(d); [23]).

## Results

Table 3 demonstrates the enhanced performance of our RAG-equipped GPT-4 models in COVID-19 fact-checking. The *P* value from the confusion matrix, used to evaluate if the model’s performance significantly differs from random guessing, was <.001 for all cases (Multimedia Appendix 2). The integration of the RAG system notably improved the LLM’s performances on the real-world dataset. Specifically, the baseline GPT-4 model achieved an accuracy of 0.856, which increased to 0.946 with the implementation of the naïve RAG system. Further improvements were observed with the LOTR-RAG model reaching an accuracy of 0.951. The CRAG and SRAG models achieved even higher accuracies of 0.972 and 0.973, respectively. These results underscore the consistent and substantial accuracy gains achieved by each RAG-equipped model, bringing their performance nearly in line with the ground-truth labels.

**Table 3:** Results of COVID-19 fact-checking.

Target dataset and models		Accuracy	*F*_1_-score	PPV^a^	Sensitivity
**Real-world dataset**					
	GPT-4	0.856	0.849	0.894	0.894
	Naïve RAG^b^	0.946	0.945	0.970	0.920
	LOTR-RAG^c^	0.951	0.952	0.959	0.944
	CRAG^d^	0.972	0.972	0.980	0.965
	SRAG^e^	0.973	0.974	0.968	0.980
**Synthesized dataset**					
	GPT-4	0.960	0.961	0.942	0.980
	Naïve RAG	0.972	0.973	0.947	1.000
	LOTR-RAG	0.978	0.978	0.965	0.992
	CRAG	0.978	0.978	0.972	0.984
	SRAG	0.978	0.978	0.969	0.988

^a^PPV: positive predictive value.

^b^RAG: retrieval-augmented generation.

^c^LOTR-RAG: Lord of the Retrievers

^d^CRAG: corrective retrieval-augmented generation.

^e^SRAG: self–retrieval-augmented generation .

The RAG system similarly enhances LLM’s performance on the synthesized dataset. The baseline GPT-4 model achieved an accuracy of 0.960, which improved to 0.972 with the naïve RAG system. The LOTR-RAG, CRAG, and SRAG models all attained an accuracy of 0.978. These results confirm that the RAG-equipped models consistently achieve high accuracy levels, closely mirroring the ground truth, and demonstrate the system’s robust performance across different datasets.

Table 4 illustrates two examples of RAG systems in enhancing COVID-19 fact-checking accuracy. The baseline GPT-4 model struggled with identifying the facts behind COVID-19 claims, often producing hallucinations. Integrating naïve RAG significantly improved the model’s performance by providing more accurate and contextually relevant responses backed by an external context dataset (ie, academic papers). Further improvements were observed with the LOTR-RAG system, which offered even more precise explanations. The CRAG and SRAG models excelled, delivering the most detailed and specific responses among all models, demonstrating the advanced RAG systems’ superiority in generating accurate and reliable COVID-19 fact-checking outputs.

**Table 4:** Examples of COVID-19 fact-checking.

Model	Response to real claim: hydroxychloroquine not safe or effective for preventing or treating COVID-19 (true)	Response to false claim: scientists confirmed COVID-19 was transmitted directly from pangolins to humans (false)
GPT-4	False	False
Naïve RAG^a^	True, several studies and clinical trials have investigated hydroxychloroquine's use for COVID-19, with varying results on its efficacy and safety.	False. While pangolins were initially considered as potential intermediate hosts, it is generally accepted that SARS-CoV-2 has a probable bat origin, and the exact pathway to human transmission remains undetermined.
CRAG^b^	Hydroxychloroquine is not effective in preventing or treating COVID-19, according to multiple studies. These include “Hydroxychloroquine or Chloroquine for Treatment or Prophylaxis of COVID-19: A Living Systematic Review” by A. V. Hernandez et al. 2020, and “A Randomized Trial of Hydroxychloroquine as Postexposure Prophylaxis for Covid-19” by D. R. Boulware et al. 2020. Despite its effectiveness in treating other conditions, it fails to prevent COVID-19 in exposed individuals or treat the disease.	Based on the given context, the claim is true. The potential for another wave of COVID-19 infections is anticipated by various disease experts, due to new variants emerging. This is referenced in the documents “Predicting the development trend of the second wave of COVID-19 in five European countries” by J. Lei, M. Li, X. Wang and “The Impact of the COVID-19 Pandemic on Health-Care Delivery System for Other Diseases and Antimicrobial Resistance Threat in India” by D. Chakraborty, A. Majumdar, F. Debnath, S. Naskar, and S. Dutta. The specifics of the potential wave, including its scale and severity, are still uncertain.
LOTR-RAG^c^	True. Based on the context provided, there is no scientific evidence to support the claim that hydroxychloroquine cures COVID-19 or is used for radiation sickness. The document titled “It is time to drop hydroxychloroquine from our COVID-19 armamentarium” by T. Kashour and I. M. Tleyjeh indicates that subsequent randomized trials revealed a lack of efficacy of hydroxychloroquine in improving the outcomes of COVID-19.	False. No definitive evidence directly links pangolins as the intermediate host transmitting SARS-CoV-2 to humans. According to “COVID-19—Zoonosis or Emerging Infectious Disease?” by N. Haider et al., 2020, no cases of natural transmission from wild or domesticated animals have been confirmed as of their study, and SARS-CoV-2 does not meet the WHO definition of zoonoses.
SRAG^d^	The claim that Hydroxychloroquine is not safe or effective for preventing or treating COVID-19 is TRUE. This is supported by a systematic review and meta-analysis conducted by Tanni et al (2021), which states that the use of Hydroxychloroquine increased the risk of adverse events and its use for prophylaxis of SARS-CoV-2 infection or treatment of mild COVID-19 is not recommended. Furthermore, M. Zepperi in 2023 warned of the drug's potential to cause retinal toxicity, leading to vision impairment or loss.	The claim that COVID-19 was directly transmitted to humans from pangolins is not confirmed. The context from the document “Analysis of the hosts and transmission paths of SARS-CoV-2 in the COVID-19 outbreak” by R. Dong et al suggests a possible transmission path from bats to pangolins to humans, but does not confirm direct transmission to humans from pangolins. The genetic analysis indicates a close relationship, implying an intermediary host, but it does not establish direct transmission to humans.

^a^RAG: retrieval-augmented generation.

^b^CRAG: corrective retrieval-augmented generation.

^c^LOTR-RAG: Lord of the Retrievers

^d^SRAG: self–retrieval-augmented generation .

In addition, Table 5 presents failure cases of our agentic RAG systems. While there are no instances of false fact-checking, some cases show the RAG system cannot find answers or retrieve mixed results. When relevant information cannot be retrieved, the system responds with “I don’t know” instead of fabricating answers. If mixed results are found, the system reports them accordingly. Therefore, CRAG and SRAG help users access relevant information and exercise caution with unclear claims, even if the full fact-checking process cannot be completed. We will discuss the limitations of the RAG-enhanced fact-checking system in the Discussion section.

**Table 5:** Illustrative cases of fact-checking failures in an agentic RAG^a^.

RAG system	Claim (ground truth)	Response
CRAG^b^	Use of hydroxychloroquine alone as a treatment for COVID-19 is associated with a higher risk of death. (true)	The claim is not unequivocally true or false since the studies show mixed results. S Arshad et al’s “Treatment with hydroxychloroquine, azithromycin, and combination in patients hospitalized with COVID-19” suggests that hydroxychloroquine alone might reduce COVID-19 mortality. Conversely, A H Elshafie et al in “Ivermectin role in COVID-19 treatment (IRICT): single-center, adaptive, randomized, double-blind, placebo-controlled, clinical trial” found that hydroxychloroquine increased the 3-month mortality rates. Finally, a third document indicates that its usage potentially increases the risk of death from COVID-19. Therefore, the claim is both supported and contradicted depending on the referred study.
CRAG	Homemade oxygen devices cannot help patients with COVID-19 at home. (false)	The claim that homemade oxygen devices cannot help patients with COVID-19 at home is too broad, as the context does not provide information specifically about homemade oxygen devices. However, Dr Connolly confirms in a paper published by *The Lancet Digital Health* that pulse oximeters can be a helpful tool for self-monitoring oxygen levels in individuals with mild COVID-19 at home. Homemade oxygen devices were not discussed in the provided context, thus a definitive statement about their effectiveness cannot be made. Title: “Effectiveness and safety of pulse oximetry in remote patient monitoring of patients with COVID-19: a systematic review.” Author: *The Lancet Digital Health*, 2022.
SRAG^c^	Nonabsorptive material has been shown to be better than absorptive material for personal protective clothing when combating coronaviruses other than SARS-CoV-2. (false)	The claim is not mentioned in the provided context. The context from the document “Efficacy of cloth face mask in reducing COVID-19 transmission: A literature review” by Rizki and Kurniawan only discuss the efficacy of cloth face masks compared with surgical masks in the context of COVID-19, but does not compare nonabsorptive and absorptive materials.
SRAG	The biological ability of bats to detect cytoplasmic DNA in their bodies helps them respond to and coexist with viruses. (true)	I don’t know. The provided context does not contain information related to the ability of bats to identify intracellular DNA or their efficiency in responding to viruses.

^a^RAG: retrieval-augmented generation.

^b^CRAG: corrective retrieval-augmented generation.

^c^SRAG: self–retrieval-augmented generation.

## Discussion

### Principal Findings

This study investigates the effectiveness of leveraging a RAG system to improve the accuracy and reliability of COVID-19 fact-checking using LLMs. Our method addresses two critical challenges: the inaccuracies and hallucinations often produced by LLMs and the high costs associated with traditional human-led fact-checking or fine-tuning methods. By integrating a vast dataset of approximately 130,000 peer-reviewed papers with GPT-4 and using an agentic RAG chain, we demonstrated significant improvements in both the accuracy and contextual relevance of the fact-checking process. Meanwhile, all of the RAG systems demonstrate cost efficiency, maintaining a low computational expense of less than US $0.08 per query in real-time applications.

The spread of myths and disinformation during the COVID-19 pandemic has been a significant challenge, exacerbating public health crises by undermining trust in scientific expertise and promoting harmful behaviors [24]. Traditional fact-checking methods, while effective, are often slow and resource-intensive, leading to delays in countering misinformation [25]. Furthermore, the sheer volume of false information circulating on the internet makes it nearly impossible for human-led efforts to keep pace [26]. Traditional approaches, such as using machine learning models to detect misinformation [5,27,28] or fine-tuning LLMs to classify tweets into categories such as “entailment,” “neutral,” and “contradiction” [29], have shown some promise. However, these methods often fall short when faced with the nuances of natural language and the complex reasoning required to debunk false claims [30].

The integration of RAG systems with LLMs represents a transformative step forward in automated fact-checking. RAG-enhanced models address these limitations by combining the retrieval of relevant information from vast corpora with the ability to contextualize them, providing accurate and easy-to-understand explanations during critical events, such as the United States presidential election [31]. Building on this progress, our RAG system adapts and extends these capabilities to meet the rigorous demands of health care and public health domains. These fields require exceptional factual accuracy and scientifically grounded explanations to ensure reliability and trust. By leveraging peer-reviewed papers as an external knowledge source, our system achieves state-of-the-art performance in health fact-checking. Furthermore, it offers a practical, cost-effective solution compared with resource-intensive alternatives, such as fine-tuned LLMs. This makes our approach both highly efficient and easily deployable for critical real-world applications.

The implications of our findings are multifaceted. First, the ability of RAG-equipped models to deliver high-accuracy fact-checking in real time could transform how misinformation is managed during public health emergencies. In response to health communication policies aimed at combating health misinformation [32], our RAG-enhanced LLMs can function as web applications or browser plugins deployed across various platforms, including social media and government websites, to provide timely and reliable information to the public. Furthermore, reducing the cost and time associated with fact-checking could enable wider adoption of these technologies, particularly in low-resource settings where misinformation can have devastating consequences. Our study highlights the potential for using advanced artificial intelligence (AI) systems to combat misinformation and improve public health literacy more broadly. The ability of our models to generate detailed, evidence-based explanations means that they could be used as educational tools, helping individuals to better understand complex health information and make informed decisions. This could be particularly valuable in addressing vaccine hesitancy and other public health challenges where misinformation plays a central role.

Our findings highlight the potential of AI-driven technologies in combating misinformation during pandemics and other public health crises. The success of the RAG-equipped LLMs in enhancing the accuracy and efficiency of COVID-19 fact-checking suggests that similar approaches could be adapted and refined for broader applications, including emerging infectious diseases, cancer, cardiovascular health, and dietary behaviors [33,34]. Future research should explore the effectiveness of knowledge-graph RAG systems and the comparative performance of fine-tuned versus RAG-equipped LLMs in enhancing automated health fact-checking. The modularity and scalability of RAG systems present opportunities for developing flexible tools tailored to specific misinformation challenges, such as vaccine safety, treatment efficacy, or preventive measures [35]. Integrating these systems into digital platforms, such as social media, news outlets, and public health websites, could provide real-time, accurate information directly to the public, countering misinformation before it gains traction [36]. Collaboration among AI developers, public health experts, and the integration of human feedback through a “human-in-the-loop” approach will be crucial for ensuring the ethical implementation and effectiveness of those technologies, fostering more informed and resilient societies during public health crises [37].

### Limitations

Despite their advantages, RAG-based LLM systems have several limitations. The external knowledge dataset derived from peer-reviewed journal papers cannot capture the entirety of academic research on COVID-19 due to limitations such as language restrictions, geographical diversity, and the lack of timely clinical insights that remain unpublished. While RAG-enhanced models improve accuracy, they are not immune to errors and biases. The quality and diversity of the academic papers directly impact the outputs of fact-checking. Misinformation generally evolves faster than peer-reviewed literature, limiting the system’s responsiveness to emerging falsehoods, which can be a limitation for real-time applications. Furthermore, the opaque nature of RAG’s decision-making reduces interpretability. Finally, the system’s effectiveness in multilingual and low-resource settings remains limited, potentially exacerbating disparities in combating health misinformation. Addressing these challenges is essential for ensuring the reliability, scalability, and equitable application of RAG-LLM fact-checking in public health.

### Conclusion

This study demonstrates that the integration of RAG systems with LLMs substantially improves the accuracy and contextual relevance of automated fact-checking. By addressing the challenges of LLM inaccuracies and the high costs of traditional fact-checking methods, our RAG-enhanced approach improves the factual correctness of outputs. It provides contextually rich explanations that can be widely applied in combating misinformation during public health crises.

## Appendix

Multimedia Appendix 1 Claims for evaluation.

Multimedia Appendix 2 Detailed results of evaluation.

## Abbreviations

AI: artificial intelligence
CRAG: corrective retrieval-augmented generation
LLM: large language model
LOTR: Lord of the Retrievers
RAG: retrieval-augmented generation
SRAG: self–retrieval-augmented generation

## Data Availability

All relevant data and code used in this study are publicly available at GitHub [38].
