# Supplementary material for: Use of Retrieval-Augmented Large Language Model for COVID-19 Fact-Checking: Development and Usability Study
Source: J Med Internet Res. 2025 Apr 30;27:e66098. doi: 10.2196/66098 (PMC12079058; doi:10.2196/66098)
Supplement: Multimedia Appendix 2 [file jmir_v27i1e66098_app2.pdf]

Results of COVID-19 Fact-checking

Target Dataset	Model	Accuracy	t-value	F1 score	t-value	PPV	t-value	Sensitivity	t-value	*P*-value
Real-world dataset	GPT-4	0.856	35.600	0.849	34.900	0.894	39.400	0.894	39.400	<.001
	Naive RAG	0.946	44.600	0.945	44.500	0.970	47.000	0.920	42.000	<.001
	LOTR-RAG	0.951	45.100	0.952	45.200	0.959	45.900	0.944	44.400	<.001
	CRAG	0.972	47.200	0.972	47.200	0.980	48.000	0.965	46.500	<.001
	SRAG	0.973	47.300	0.974	47.400	0.968	46.800	0.980	48.000	<.001
Synthesized dataset	GPT-4	0.960	46.000	0.961	46.100	0.942	44.200	0.980	48.000	<.001
	Naive RAG	0.972	47.200	0.973	47.300	0.947	44.700	1.000	50.000	<.001
	LOTR-RAG	0.978	47.800	0.978	47.800	0.965	46.500	0.992	49.200	<.001
	CRAG	0.978	47.800	0.978	47.800	0.972	47.200	0.984	48.400	<.001
	SRAG	0.978	47.800	0.978	47.800	0.969	46.900	0.988	48.800	<.001

*The p-value derived from a confusion matrix is a statistical significance measure that assesses whether the model performances differ significantly from what would be expected under random guessing.
